# Neuroprotective mechanisms of puerarin in middle cerebral artery occlusion-induced brain infarction in rats

**DOI:** 10.1186/1423-0127-16-9

**Published:** 2009-01-19

**Authors:** Yi Chang, Cheng-Ying Hsieh, Zi-Aa Peng, Ting-Lin Yen, George Hsiao, Duen-Suey Chou, Chien-Ming Chen, Joen-Rong Sheu

**Affiliations:** 1 Department of Anesthesiology, Shin Kong Wu Ho-Su Memorial Hospital and School of Medicine, Fu-Jen Catholic University, Taipei; 2 Graduate Institute of Medical Sciences, Taipei Medical University, Taipei 110, Taiwan; 3 Department of Pharmacology, Taipei Medical University, Taipei 110, Taiwan

## Abstract

Puerarin, a major isoflavonoid derived from the Chinese medical herb *Radix puerariae *(kudzu root), has been reported to be useful in the treatment of various cardiovascular diseases. In the present study, we examined the detailed mechanisms underlying the inhibitory effects of puerarin on inflammatory and apoptotic responses induced by middle cerebral artery occlusion (MCAO) in rats. Treatment of puerarin (25 and 50 mg/kg; intraperitoneally) 10 min before MCAO dose-dependently attenuated focal cerebral ischemia in rats. Administration of puerarin at 50 mg/kg, showed marked reduction in infarct size compared with that of control rats. MCAO-induced focal cerebral ischemia was associated with increases in hypoxia-inducible factor-1α (HIF-1α), inducible nitric oxide synthase (iNOS), and active caspase-3 protein expressions as well as the mRNA expression of tumor necrosis factor-α (TNF-α) in ischemic regions. These expressions were markedly inhibited by the treatment of puerarin (50 mg/kg). In addition, puerarin (10~50 μM) concentration-dependently inhibited respiratory bursts in human neutrophils stimulated by formyl-Met-Leu-Phe. On the other hand, puerarin (20~500 μM) did not significantly inhibit the thiobarbituric acid-reactive substance reaction in rat brain homogenates. An electron spin resonance (ESR) method was conducted on the scavenging activity of puerarin on the free radicals formed. Puerarin (200 and 500 μM) did not reduce the ESR signal intensity of hydroxyl radical formation. In conclusion, we demonstrate that puerarin is a potent neuroprotective agent on MCAO-induced focal cerebral ischemia *in vivo*. This effect may be mediated, at least in part, by the inhibition of both HIF-1α and TNF-α activation, followed by the inhibition of inflammatory responses (i.e., iNOS expression), apoptosis formation (active caspase-3), and neutrophil activation, resulting in a reduction in the infarct volume in ischemia-reperfusion brain injury. Thus, puerarin treatment may represent a novel approach to lowering the risk of or improving function in ischemia-reperfusion brain injury-related disorders.

## Background

Puerarin (daidzein-8-C-glucoside), is the major isoflavonoid derived from the Chinese medical herb *Radix puerariae *(kudzu root). In China, *R. puerariae *is known as *Ge Gen*, and has been used as a traditional medicine for treating various diseases including cardiovascular disorders [1]. Puerarin has been shown to be effective in treating heart diseases such as angina and myocardial infarction [2]. The protective mechanisms of puerarin, at least in part, are related to its ability to increase superoxide dismutase activity, decrease lipid peroxidation, and enhance fibrinolysis [2,3]. Furthermore, puerarin can also increase cerebral blood flow in dogs [4], and reduces cerebral and spinal cord injury in ischemia-reperfusion rats and rabbits [5,6]. Several studies have also revealed that puerarin possesses protective effects against cortical neuron and astrocyte damage induced by oxygen-glucose deprivation or glutamate excitotoxicity *in vitro *[7].

Ischemic hypoxic brain injury often causes irreversible brain damage. The cascade of events leading to neuronal injury and death in ischemia includes the release of cytokines and free radicals, and induction of inflammation, apoptosis, and excitotoxicity [8]. Reperfusion of ischemic areas could exacerbate ischemic brain damage through the generation of reactive oxygen species (ROS), including superoxide anions (O_2_^•-^), hydroxyl radicals (OH^•^), and nitric oxide (NO) [9]. Neutrophils are a potential source of ROS when activated during inflammatory responses [10]. When a tissue suffers from ischemia and reperfusion, proinflammatory cytokines produced by inflammatory cells can trigger adhesion and migration of circulating neutrophils to endothelial cells and generation of ROS that enhances neutrophil infiltration and results in ischemic injury [11]. Furthermore, ROS also mediate a mitochondrial signaling pathway that may lead to apoptosis followed by cerebral ischemia [12]. Various *in vitro *studies have demonstrated that cellular or biochemical signaling pathways involve mitochrondria-derived activator of caspases, activation of downstream caspase-9 and -3, and DNA fragmentation [12]. Therefore, pharmacological agents which reduce ROS formation have been found to limit the extent of brain damage following stroke-like events [13].

Recently, Xu *et al. *[7] demonstrated that puerarin (100 mg/kg) possesses neuroprotective effects against cerebral ischemia in rats. However, the detailed mechanisms underlying the neuroprotective effects of puerarin in inflammatory and apoptotic responses induced by middle cerebral artery occlusion (MCAO) have still not yet been completely resolved. We therefore further examined the effect of puerarin in MCAO-induced cerebral ischemia, and utilized the findings to further characterize the neuroprotective mechanisms of puerarin.

## Methods

### Materials

Puerarin, 2,3,5-triphenyltetrazolium (TTC), aprotinin, cremophor EL, leupeptin, lucigenin, N-formyl-Met-Leu-Phe (fMLP), and bovine serum albumin (BSA) were purchased from Sigma (St. Louis, MO). Ficoll-Paque plus was purchased from Amersham (Buckinghamshire, HP, UK). Puerarin was dissolved in solvent (cremophor: ethanol: normal saline at 1: 1: 4) for the in vivo studies, and dissolved in 0.5% DMSO for the in vitro studies.

### MCAO-induced transient focal cerebral ischemia in rats

Male Wistar rats (250~300 g) were used in this study. All animal experiments and care were performed according to the Guide for the Care and Use of Laboratory Animals (National Academy Press, Washington, DC, 1996). Before undergoing the experimental procedures, all animals were clinically normal, were free of apparent infection or inflammation, and showed no neurological deficits.

Animals were anesthetized with a mixture of 75% air and 25% O_2 _gases containing 3% isoflurane. The rectal temperature was maintained at 37 ± 0.5°C. The right middle cerebral artery (MCA) was occluded as described in our previous report [14]. Briefly, the right common carotid artery was exposed, and a 4-0 monofilament nylon thread (25 mm) coated with silicon was then inserted from the external into the internal carotid artery until the tip occluded the origin of the MCA. After closure of the operative sites, the animals were allowed to awake from the anesthesia. During another brief period of anesthesia, the filament was gently removed after 1 h of MCAO. An observer blinded to the identity of the groups assessed the neurological deficits at 1 and 24 h after reperfusion (before euthanization) by the forelimb akinesia (also called the postural tail-hang) test, whereas the spontaneous rotational test was used as a criterion for evaluating the ischemic insult [15]. Animals not showing behavioral deficits at the above time points after reperfusion were excluded from the study. On the other hand, reperfusion was also ensured by an improvement in ipsilateral local blood flow to at least 60% of the baseline following an initial sharp decrease to about 50–60% of the baseline caused by MCAO as determined using a continuous laser Doppler flowmeter (LDF; Oxford Array™, Oxford Optronix, Oxford, UK) with a standard needle probe (pp-051). For the continuous measurement of local regional cerebral blood flow (rCBF), the rat was fixed in a stereotaxic frame. The skull was exposed, and a burr hole in a diameter of 2 mm was drilled on the right side of the skull at 2 mm posterior and 5 mm lateral to the bregma to accommodate the probeholder. LDF was used to estimate blood flow continuously during ischemia and reperfusion.

Rats were euthanized by decapitation after 24 h of reperfusion. The brains were cut into 2-mm coronal slices starting 1 mm from the frontal pole. Each stained brain (2% TTC) slice was drawn using a computerized image analyzer (Image-Pro plus). The calculated infarct areas were then compiled to obtain the infarct volume (mm^3^) per brain. Infarct volumes were expressed as a percentage of the contralateral hemisphere volume using the formula: (the area of the intact contralateral [left] hemisphere – the area of the intact region of the ipsilateral [right] hemisphere) to compensate for edema formation in the ipsilateral hemisphere [14].

All animals were divided into four groups: (1) a sham-operated group; (2) a solvent-treated (solvent) group (cremophor: ethanol: normal saline at 1: 1: 4); and groups treated with a single dose of (3) 25 or (4) 50 mg/kg, i.p. of puerarin. In the group treated with the solvent or puerarin, rats were given the isovolumetric solvent or puerarin (25 or 50 mg/kg) 10 min before MCAO.

### Neurobehavioral test

The sensorimotor integrity was conducted to assess the neurobehavior at 1 and 24 h after MCAO in rats [15]. Five categories of motor neurological findings were scored: 0, no observable deficit; 1, forelimb flexion; 2, forelimb flexion and decreased resistance to lateral push; 3, forelimb flexion, decreased resistance to lateral push and unilateral circling; 4, forelimb flexion, unable or difficult to ambulate.

### Determination of the expressions of HIF-1α, iNOS, and active caspase-3 in MCAO-insulted brain

The expressions of HIF-1α, iNOS, and active caspase-3 in the brain at 24 h after MCAO-reperfusion injury were analyzed by Western blotting as described by Rodrigo *et al. *[16] with minor modifications. The MCAO-insulted and sham-operated rats were anesthetized with chloral hydrate (400 mg/kg, i.p.), then the apex of the heart was penetrated with a perfusion cannula inserted through the left ventricle into the ascending aorta. Perfusion with ice-cold phosphate-buffered saline (PBS) was performed, and an incision was made in the right atrium for venous drainge. Brains were removed freshly and sectioned coronally into four sequential parts from the frontal lobe to the occipital lobe. The third parts of the right hemisphere was separately collected, snap-frozen in liquid nitrogen, and stored at -70°C. The frozen tissues were placed in homogenate buffer and homogenized, then sonicated for 10 s three times at 4°C. The sonicates were subjected to centrifugation (10,000 *g*).

The supernatant (50 μg protein) was subjected to SDS-PAGE and electrophoretically transferred to PVDF membranes (0.45 μm; Hybond-P; Amersham). After incubation in blocking buffer and being washed three times with TBST buffer (10 mM Tris-base, 100 mM NaCl, and 0.1% Tween 20; pH 7.5), the blots were treated with an anti-HIF-1α polyclonal antibody (pAb, 1: 1000; R&D, Minneapolis, MN), an anti-iNOS monoclonal antibody (mAb; 1: 3000, BD Biosciences, San Jose, CA), and an anti-active caspase-3 pAb (1: 250; Biovision, Mountain View, CA), or an anti-α-tubulin mAb (1: 2000; Santa Cruz Biotech, Santa Cruz, CA) in TBST buffer overnight. Blots were subsequently washed with TBST and incubated with secondary horseradish peroxidase-conjugated goat anti-mouse mAb or donkey anti-rabbit IgG (Amersham) for 1 h. The blots were then washed, and the immunoreactive protein was detected using film exposed to enhanced chemiluminescence detection reagents (ECL^+ ^system; Amersham). The bar graph depicts the ratios of quantitative results obtained by scanning reactive bands and quantifying the optical density using Videodensitometry (Bio-1D version 99 image software).

### Isolation of total RNA and reverse-transcription polymerase chain reaction (RT-PCR)

Fresh brains were removed, separated into ipsilateral and contralateral hemispheres, snap frozen in liquid nitrogen and stored at -70°C until used as stated above. Total RNA was isolated from the ipsilateral cortex by a commercially available kit according to the manufacturer's instructions (TRIzol, Gibco). For each RT-PCR, 0.5 mg of the RNA sample and 0.2 μM of primers were reverse-transcribed and amplified in a 50-μl reaction mixture of commercially available reagents (SUPERSCRIPT One-Step RT-PCR with PLATINUM Taq Kit, Invitrogen) containing a 1× reaction mixture and 0.2 μM of an RT/Taq mixture in one cycle of 30 min at 50°C for reverse transcription and one cycle at 95°C for 3 min, followed by 40 cycles at 95, 62, and 72°C for 30, 40, and 40 s, respectively; with a single extension step at 72°C for 5 min followed by 4°C for amplification in a thermal cycler (GeneAmp PCR system 2400, Perkin-Elmer). For visualization and quantification by densitometry of each RT-PCR, a 10-μl aliquot was subjected to electrophoresis on a 1.5% agarose gel using a mini horizontal submarine unit (HE 33) containing 0.5 mg/ml ethidium bromide to allow UV-induced fluorescence (TCP-20.M, Vilber Lourmat). Preliminary experiments were performed to determine the range of amplification cycles and the beginning RNA substrate within the linear phase of the exponential increase of the PCR products for each particular primer pair.

### Determination of respiratory bursts in human neutrophils

Superoxide anion production of neutrophils was measured by the method of lucigenin-enhanced chemiluminescence (LCL) as described by Hsiao *et al. *[17] with some modifications. Washed neutrophil suspensions (2 × 10^6 ^cells/ml) in modified Hank's balanced salt solution (HBSS) containing 1 mM CaCl_2 _and 0.5 mM MgCl_2 _were dispensed into wells of a standard scintillation microplate. Before the assay, cells were preincubated with the solvent control (0.5% DMSO) or various concentrations of puerarin (10, 20, and 50 μM). Then, 20-μl aliquots of lucigenin were added at a final concentration of 100 μM. The basal LCL was recorded for 1 min by a microplate luminometer (Orion^®^, Berthold, Germany) at 37°C, and cells were immediately stimulated with fMLP (800 nM). The luminescent light was continuously recorded for 5 min. The chemiluminescent signal was represented as relative light units per second (RLU/s). The results of LCL intensity (as increments in the signal intensity) were determined by measuring basal and stimulator-induced peak values and calculating the difference between them.

### Antioxidative activity of puerarin in rat brain homogenate preparations

Rat brain homogenates were prepared from brains of freshly killed Wistar rats, and the peroxidation in the presence of iron ions was measured by the thiobarbituric acid (TBA) method, as described by Braughler *et al. *[18] with some modifications. In brief, whole brain tissue, excluding the cerebellum, was washed and homogenized in 10 volumes of ice-cold Krebs buffer. The homogenate was centrifuged, and the supernatant was used immediately for the lipid peroxidation assays. The reaction mixture with puerarin or vehicle solution (0.5% DMSO) was incubated for 10 min, then stimulated by the addition of ferrous ions (200 μM). The reactions were terminated by adding a trichloroacetic acid solution and the TBA-reactive substance (TBARS) reagent. After boiling for 15 min, samples were cooled and extracted with *n*-1-butanol. The extent of lipid peroxidation was estimated as TBARS and was read at 532 nm in a spectrophotometer (Hitachi, Model U3200). Tetramethoxypropane was used as a standard, and the results were expressed as nanomoles of malondialdehyde equivalents per milligram protein of the supernatant of rat brain homogenates. The protein contents of the brain homogenates and other preparations were determined with the Bio-Rad method.

### Electron spin resonance (ESR) spectrometry

ESR spectra were recorded on a Bruker EMX ESR spectrometer in the H_2_O_2_/NaOH/DMSO system as described previously [13]. Briefly, 100 μl of DMSO and the same volumes of 25 mM NaOH and puerarin (200 and 500 μM) were mixed in a test tube, followed by the addition of 10 μl DMPO and 100 μl of 30% H_2_O_2_. The reaction mixture was aspirated into a quartz flat cell and set in the ESR apparatus; scanning was begun 1 min after all reagents were mixed. The rate of free radical-scavenging activity was defined by the following equation: inhibition rate = 1-[signal height (puerarin)/signal height (solvent control)] [13].

### Statistical analysis

The experimental results are expressed as the means ± S.E.M. and are accompanied by the number of observations. Student's unpaired *t*-test was used to determine significant differences in the study of MCAO-induced cerebral ischemia. The other experiments were assessed by the method of analysis of variance (ANOVA). If this analysis indicated significant differences among the group means, then each group was compared using the Newman-Keuls method. A *P *value of < 0.05 was considered statistically significant.

## Results

### Effect of puerarin on MCAO-induced focal cerebral ischemia in rats

**Figure 1:**
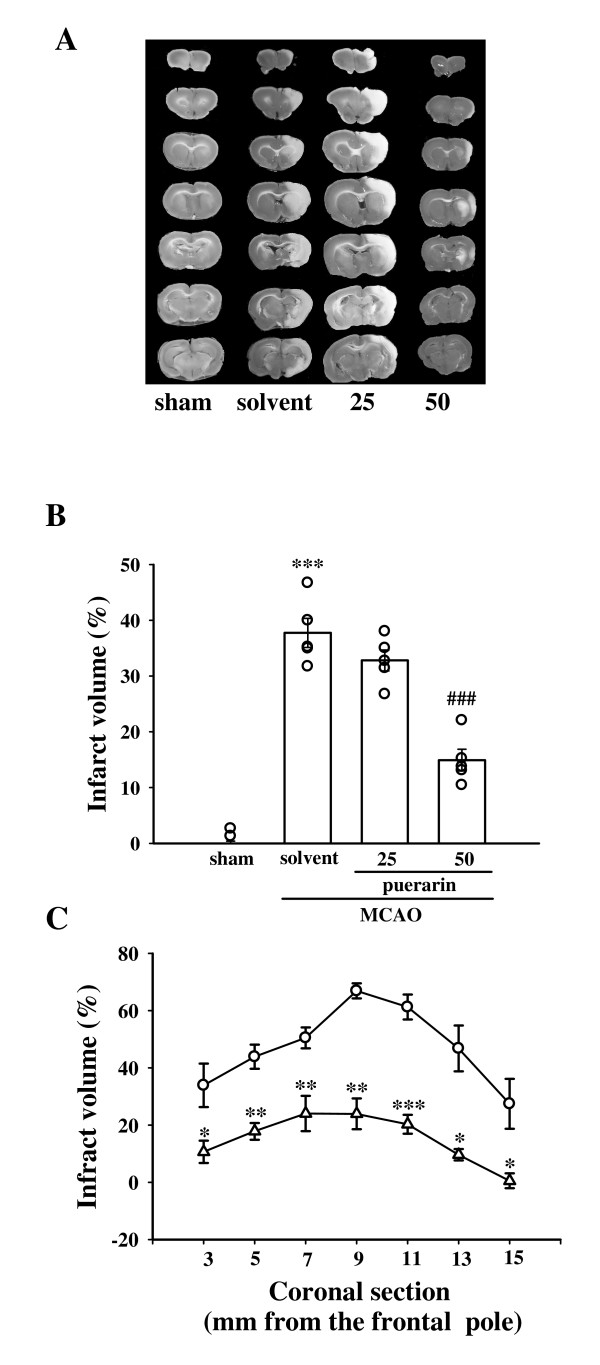
**Effect of puerarin in middle cerebral artery occlusion (MCAO)-induced focal cerebral ischemia in rats**. (A) Coronal sections of TTC-stained brains, (B) dose-response effect of puerarin, and (C) infarct area at various distances from the frontal pole in 24 h after MACO-reperfusion rats. Cerebral infarction in sham-operated (sham) or MACO-reperfusion rats is from a representative animal that received solvent (solvent; cremophor: ethanol: normal saline at 1: 1: 4) or puerarin (25 and 50 mg/kg) intraperitoneally. (B) Infarct volumes were calculated as described in "Methods", and data are presented as a superimposed scatterplot showing the infarct volume for each animal in the group as well as the means ± S.E.M. (*n *= 5). ***P < 0.001 compared to the sham-operated group; ^###^*P *< 0.001 compared to the solvent-treated group. (C) Forebrain profiles of the infarct area at various distances from the frontal pole as described in "Methods". Each point (○, solvent-treated group; △, puerarin 50 mg/kg-treated group) and vertical bar represent the means ± S.E.M. (*n *= 5). *P < 0.05, **P < 0.01, and ***P < 0.001 compared to the solvent-treated group.

**Figure 2:**
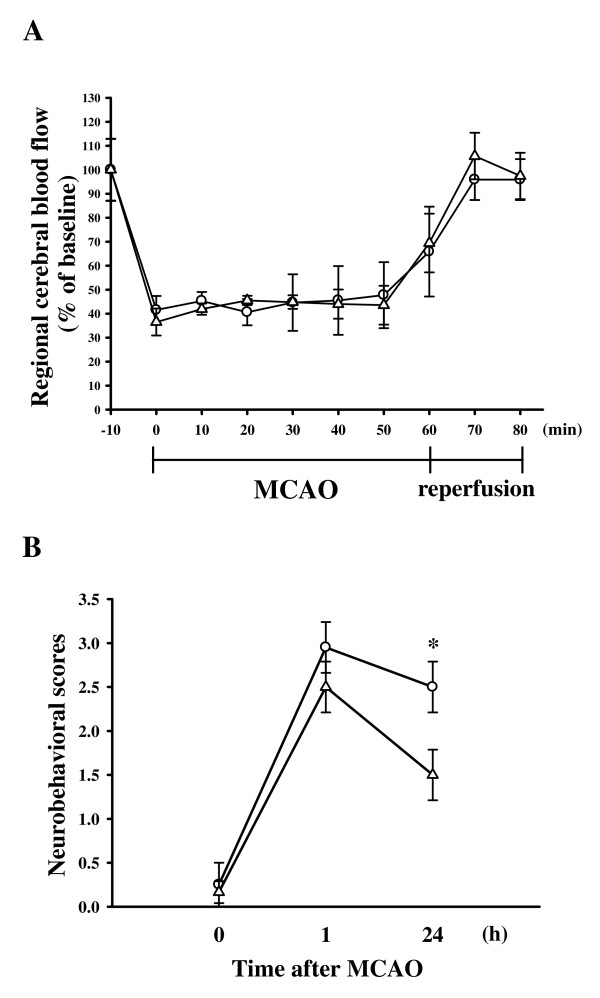
**Effect of puerarin in middle cerebral artery occlusion (MCAO)-induced regional cerebral blood flow (rCBF) and neurobehavioral deficits in rats**. (A) rCBF of solvent- and puerarin (50 mg/kg)-treated rats were measured by laser Doppler flowmeter in the MCA-supplied cortex. The value of rCBF were recorded every 10 min after MCAO and reperfusion periods. Each point (○, solvent-treated group; △, puerarin 50 mg/kg-treated group) and vertical bar represent the means ± S.E.M. (*n *= 6). (B) Neurobehavioral scores of solvent- and puerarin (50 mg/kg)-treated rats were recorded at 1 and 24 h after MCAO. Each point (○, solvent-treated group; △, puerarin 50 mg/kg-treated group) and vertical bar represent the means ± S.E.M. (*n *= 5).

All animals in this study showed similar physiological values (i.e., rectal temperature and mean arterial blood pressure) before, during, and after MCAO among groups (data not shown). Neither abnormal behavior, depression of respiration, nor hypothermia was observed in the solvent- or puerarin-treated groups. The cerebral infarction was examined using 2-mm-thick slices of the cerebrum 24 h after MCAO reperfusion in rats through TTC staining. Figure 1A shows typical photographs of coronal sections of the sham-operated, solvent (cremophor: ethanol: normal saline at 1: 1: 4)-treated, and puerarin-treated groups (25 and 50 mg/kg) prior to the ischemic insult. Administration of puerarin at 25 and 50 mg/kg showed dose-dependent reductions in infarct volume (white area) compared to the solvent-treated group (solvent, 37.7 ± 2.6% vs. 25 mg/kg, 32.8 ± 1.9%; 50 mg/kg, 14.9 ± 2.0%, *n *= 5) (Fig. 1B). On the other hand, treatment with the solvent did not significantly influence the infarct size compared with the MCAO group (without treatment with solvent or drugs) (data not shown). Figure 1C gives statistical results of the infarct areas of solvent- and puerarin (50 mg/kg)-treated groups at various distances from the frontal pole. The infarct area was largest between the 3rd and 4th sections in both groups. Treatment with puerarin (50 mg/kg) markedly reduced the infarct area in all regions, especially in sections three to five (Fig. 1C). In addition, the relative changes of regional cerebral blood flow (rCBF) were recorded using LDF during MCAO-reperfusion periods. In all groups, MCAO induced a similar immediate reduction of rCBF to approximately 50–60% of the baseline level. The rCBF value of puerarin (50 mg/kg)-treated group was not significantly influenced as compared to the solvent-treated group at the 10 min after MCAO (solvent-treated group, 95.9 ± 8.5% vs. puerarin-treated group, 105.7 ± 9.7%, *P *> 0.05; *n *= 6) (Fig. 2A). This result may indicate that the neuroprotective effect of puerarin in MCAO-induced cerebral ischemia could not relate to its ability to increase rCBF. In addition, an obvious improvement was observed in neurological function of puerarin (50 mg/kg)-treated rats at 24 h after MCAO than that of solvent-treated group (Fig. 2B).

### Effects of puerarin on HIF-1α, iNOS, and active caspase-3 protein expressions in ischemic cerebral tissues

**Figure 3:**
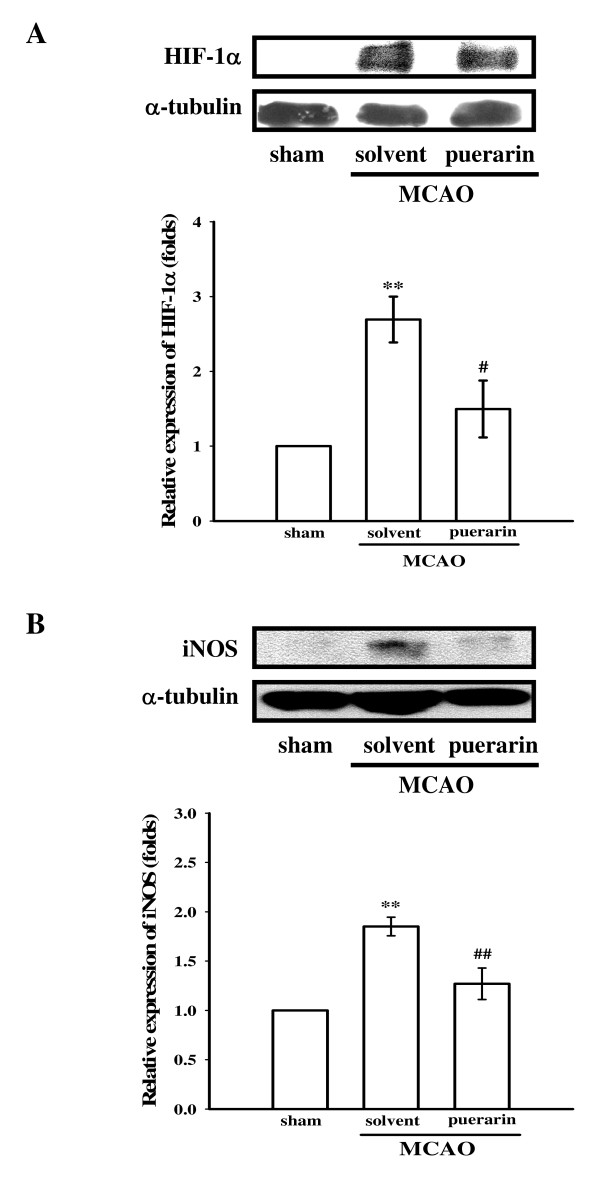
**Effects of puerarin on the expressions of (A) hypoxia-inducible factor-1α (HIF-1α) and (B) inducible nitric oxide synthase (iNOS) in cerebral homogenates 24 h after middle cerebral artery occlusion (MCAO)-reperfusion injury in rats**. Fresh brains from sham-operated (lane 1), solvent-treated (lane 2), and puerarin (50 mg/kg)-treated (lane 3) rats were removed and sectioned coronally into four sequential parts from the frontal lobe to the occipital lobe. The third part of the four sequential parts of the ischemic-injured hemisphere was separately collected, homogenized, and centrifuged. The supernatant (50 μg protein) was then subjected to SDS-PAGE, and transferred onto membranes for analysis of HIF-1α and iNOS expressions. The results are representative examples of three similar experiments. Data are presented as the means ± S.E.M. **P < 0.01 compared to the sham-operated group (lane 1); ^#^*P *< 0.05 and ^##^*P *< 0.01 compared to the solvent-treated group (lane 2). Equal loading in each lane is demonstrated by similar intensities of α-tubulin.

**Figure 4:**
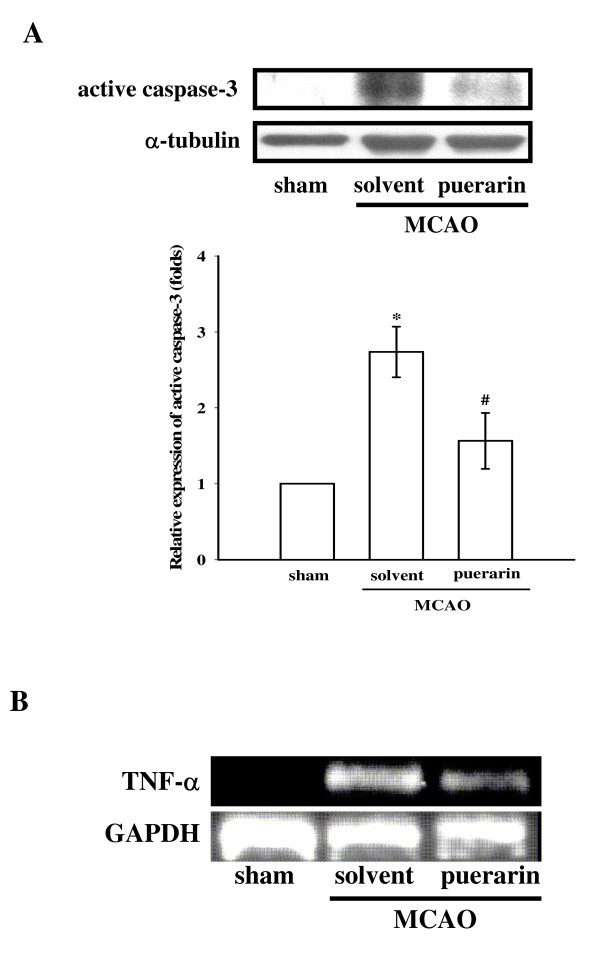
**Effects of puerarin on the expressions of (A) active caspase-3 protein and (B) tumor necrosis factor (TNF)-α mRNA in cerebral homogenates 24 h after middle cerebral artery occlusion (MCAO)-reperfusion injury in rats**. (A) Fresh brains from sham-operated, solvent-treated, and puerarin (50 mg/kg)-treated rats were removed, and sectioned coronally into four sequential parts from the frontal lobe to the occipital lobe. The third part of the four sequential parts of the ischemic-injured hemisphere was separately collected, homogenized, and centrifuged. The supernatant (50 μg protein) was then subjected to SDS-PAGE, and transferred onto membranes for analysis of active caspase-3 expression. The results are representative examples of three similar experiments. Data are presented as the means ± S.E.M. *P < 0.05 compared to the sham-operated group (lane 1); ^#^*P *< 0.05 compared to the solvent-treated groups (lane 2). Equal loading in each lane is demonstrated by similar intensities of α-tubulin. (B) Fresh brains from sham-operated, solvent-treated, and puerarin (50 mg/kg)-treated rats were removed, homogenized, and centrifuged from the ipsilateral cortex, followed by analysis of TNF-α mRNA expression by RT-PCR as described in "Methods". The GAPDH level was used to normalize the amount of the cDNA template used in each PCR reaction. The results are representative examples of three similar experiments.

Results of Western blotting of MCAO-insulted cerebral tissues are shown in Figures 3 and 4. As shown in Figure 3A, HIF-1α, detected as a major band of approximately 120 kDa 24 h after MCAO-reperfusion injury (lane 2) was more pronounced than that of levels obtained in the corresponding area of the sham-operated group (lane 1). Puerarin (50 mg/kg) treatment significantly (*P *< 0.05) suppressed the expression of HIF-1α in ischemic cerebral tissues (Fig. 3A, lane 3). In Figure 3B, the iNOS band, detected as a major band of approximately 135 kDa, showed significant increases in ischemic cerebral tissues 24 h after MCAO-reperfusion compared to that of sham-operated rats. With administration of puerarin (50 mg/kg), iNOS expression was markedly reduced in MCAO-reperfusion rats (Fig. 3B).

In addition, negative immunostaining was obtained for active caspase-3 in the sham-operated group (Fig. 4A, lane 1). At 24 h after MCAO-reperfusion, strong staining of active caspase-3 (17 kDa) was observed in ischemic cerebral tissues (lane 2) compared to levels obtained in the corresponding area of the sham-operated group (lane 1). Again, puerarin (50 mg/kg) abolished the elevation of active caspase-3 (Fig. 4A, lane 3).

### Effect of puerarin on TNF-α mRNA expression in ischemic cerebral tissues

**Figure 5:**
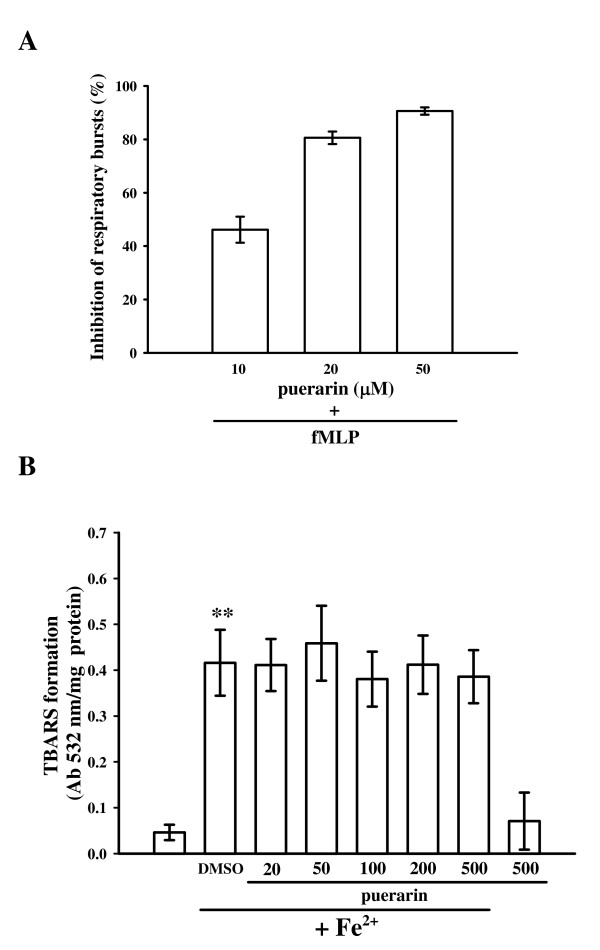
**Effects of puerarin on (A) respiratory bursts in human neutrophils, and (B) the antioxidation in thiobarbituric acid-reactive substance (TBARS) in rat brain homogenate**. (A) Washed neutrophil suspensions (2 × 10^6 ^cells/ml) were preincubated with the solvent control (0.5% DMSO) or various concentrations of puerarin (10, 20, and 50 μM) in the presence of lucigenin (100 μM), followed by the addition of fMLP (800 nM) to trigger neutrophil respiratory bursts. Data are presented as a percent inhibition of the solvent control (means ± S.E.M., *n *= 4). (B) Brain homogenates were preincubated with solvent (0.5% DMSO) or various concentrations of puerarin (20~500 μM) for 10 min followed by the addition of Fe^2+ ^(200 μM). Results are presented as the absorbance at 532 nm/mg protein in brain homogenates. Data are presented as the means ± S.E.M. (*n *= 4). **P < 0.01 compared with the solvent group (DMSO only).

Transient MCAO resulted in a significant and more-sustained increase in the expression of TNF-α mRNA in the injured hemisphere compared to levels obtained in the corresponding area of the sham-operated group (Fig. 4B). Puerarin (50 mg/kg) treatment markedly reduced this reaction (Fig. 5).

### Effects of puerarin on respiratory bursts in human neutrophils and lipid peroxidation in rat brain homogenates

The inhibitory effect of puerarin on neutrophil activation was evaluated by fMLP-induced lucigenin-dependent chemiluminescence (LCL), which is an index of respiratory bursts, as produced by superoxide anions. When human neutrophils (2 × 10^6 ^cells/ml) were treated with fMLP (800 nM), a rapid generation of superoxide anions was observed with the LCL signal. Puerarin (10~50 μM) inhibited the increase in chemiluminescence stimulated by fMLP in a concentration-dependent manner (Fig. 5A). At 50 μM, puerarin greatly reduced the LCL stimulated by fMLP to about 90% compared to the solvent control (0.5% DMSO).

In addition, puerarin was further tested for its ability to inhibit non-enzymatic lipid peroxidation in rat brain homogenates stimulated by ferrous ions. At 20~500 μM, puerarin did not significantly inhibit ferrous ions-induced lipid peroxidation in rat brain homogenates (Fig. 5B). In addition, puerarin (500 μM) did not interfere with the TBA test, since color formation did not change if it was added after incubation with TBA reagents. On the other hand, α-tocopherol (200 μM) inhibited ion-dependent lipid peroxidation in this reaction (data not shown).

### Free radical-scavenging activity of puerarin

**Figure 6:**
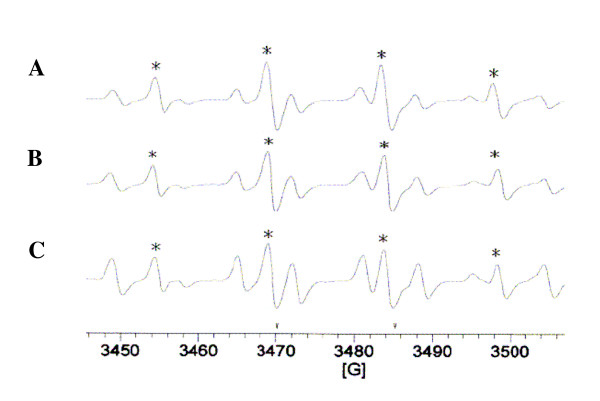
**Effect of puerarin on the free radical-scavenging activity in the H_2_O_2_/NaOH/DMSO system**. The signal of hydroxyl radical peaks was observed in electron spin resonance (ESR) experiments. (A) Typical ESR spectra in the presence of solvent control (0.5% DMSO) or puerarin (B, 200 μM), (C, 500 μM) in the H_2_O_2_/NaOH/DMSO system, the spectrum is a representative example of four similar experiments.

In this study, typical ESR signals of hydroxyl radicals were observed as shown in Figure 6. Puerarin (200 and 500 μM) did not significantly suppress hydroxyl radical formation as compared to the solvent-treated group. This observation provides *in vitro *evidence suggesting the neuroprotective effect of puerarin did not mediate by the free radical-scavenging activity.

## Discussion

The present study demonstrates that MCAO-reperfusion injury induces HIF-1α, iNOS, and active caspase-3 protein expressions, and TNF-α mRNA expression, which may represent the response of neurons suffering from ischemic insult. Animal models of focal cerebral ischemia, for which MCAO is usually used, reproduce the pattern of ischemic brain damage observed in many human ischemic stroke patients [19].

The increased HIF-1α protein level observed after MCAO-reperfusion is presumably induced by a loss of the oxygen supply [20], resulting in a greater extent of binding activity to the iNOS gene and reaching a consequent peak of iNOS protein expression. Since the iNOS gene contains the hypoxia-responsive enhancer (HRE) sequence to which HIF-1α binds [21], results from primary neuronal cultures of cells demonstrated that HIF-1α binds to the iNOS promoter gene under hypoxic conditions. Such binding is associated with an increase in iNOS expression [22]. Furthermore, HIF-1α combining with p53 may promote apoptotic cell death in ischemic areas [23]. In addition, the increased expression of iNOS may also contribute to enhanced neuronal injury, since iNOS knock-out mice show reduced brain damage after ischemia [24].

Several apoptosis-related proteins, including caspase-9 and -3, were all strongly expressed after ischemic injury. In addition, hypoxia may cause HIF-1α to bind to p53 in order to stabilize it, and also activates the expression of various genes including Bax (a proapoptotic member of the Bcl-2 family) [25]. Bax is translocated to the mitochondria where it releases cytochrome c into the cytosol to interact with Apaf-1 to activate caspase-9, which in turn activates downstream caspases, such as active caspase-3 [26]. In the present study, we showed that elevations of active caspase-3 and iNOS expressions occurred in the same time frame as HIF-1α expression after ischemic injury, and these expressions could be significantly suppressed by puerarin. In addition, TNF-α is one of the key immunomodulatory and proinflammatory cytokines upregulated during brain ischemia [27]. Administration of TNF-α during ischemic brain insult has been shown to augment the injury, as evidenced by increased tissue damage and neurological deficits [27]. In addition to inflammation, TNF-α has also been shown to be involved in apoptosis [28]. In this study, we also demonstrated that puerarin can downregulate the transcription of TNF-α during brain ischemia. Therefore, inhibition of active caspase-3 expression by puerarin may occur, at least partially, through the inhibition of TNF-α expression in ischemic brain injury.

Leukocytes, particularly neutrophils, contribute to the initiation of ischemic stroke. Thus, infiltration of neutrophils into the infarct areas following cerebral ischemia-reperfusion injury also plays a crucial role in the development of cerebral infarction and neuronal damage [11]. Although neutrophils produce and release a variety of toxic agents designed to kill microbes, those systems that depend on reactive products of oxygen metabolism are especially potent. These agents are produced as a consequence of respiratory bursts, a series of events triggered by phagocytosis or exposure to certain inflammatory mediators and feature a dramatic increase in oxidative metabolism with direct conversion of molecular oxygen to its univalent reduction product, the superoxide anion. Our results showed that puerarin significantly inhibited neutrophil respiratory bursts by the chemotactic tripeptide, fMLP, which can activate leukocytes, resulting in leukocyte adhesion to the endothelium, leukocyte aggregation, decreased leukocyte deformability, and the release of oxidants, proteases, and lipid metabolites [29].

The phospholipid bilayers of cellular and subcellular membranes are undoubtedly major targets for free radicals. The compound that inhibits membrane phospholipid peroxidation seems to exert a pharmacological effect in preventing radical-induced oxidative pathological events. Among cell-free systems, brain homogenates are usually chosen to evaluate an antioxidant's effect on lipid peroxidation [18]. Rat brain homogenates exposed to ferrous ions exhibit lipid peroxidation in air by a mechanism whose induction step may primarily involve site-bound, iron-mediated decomposition of lipid hydroperoxides to yield alkoxy or peroxyl radicals, leading to the chain reaction of lipid peroxidation [30]. In this system, puerarin did not effectively inhibit lipid peroxidation, indicating that the inhibition of lipid peroxidation might not be the neuroprotective mechanism of puerarin in ischemic brain injury.

In conclusion, the most important findings of this study suggest that the neuroprotective effect of puerarin on cerebral ischemic damage in MCAO-reperfusion rats is probably mediated by the inhibition of HIF-1α and TNF-α, followed by the inhibition of inflammatory responses (i.e., iNOS), apoptosis (active caspase-3), and neutrophil activation. Puerarin itself possesses neither free radical-scavenging nor antioxidative activity, whereas it may reduce superoxide anion formation probably through inhibiting neutrophil activation. The rationale for the use of puerarin is based on the fact that multiple deleterious processes in different cell types of organelles are initiated during ischemia-reperfusion injury which ultimately synergistically moves toward irreversible injury. Therefore, treatment using puerarin is not limited to one factor but involves many mechanisms, most of which may be interrelated. For example, puerarin-induced neuroprotection is related to inflammation, NO, and apoptosis, and many of those factors (such as iNOS, active caspase-3, etc) are related to HIF-1α. We speculate that the correction of these molecules and morphological changes may lead to neurobehavioral improvement in patients; thus, treatment using puerarin may represent an ideal approach for improving function after ischemia-reperfusion brain injury.

## Competing interests

The authors declare that they have no competing interests.

## Authors' contributions

YC, CYH, and ZAP carried out the animal study. YC, TLY, and GH participated in the Western blotting assays. GH carried out the neutrophil study. DSC and CMC carried out the ESR study. YC and CYH participated in the design of the study and performed the statistical analysis. JRS conceived of the study, participated in its design and coordination, and collectively prepared the manuscript. All authors read and approved the final manuscript.
